# A nine-hub-gene signature of metabolic syndrome identified using machine learning algorithms and integrated bioinformatics

**DOI:** 10.1080/21655979.2021.1968249

**Published:** 2021-09-13

**Authors:** Guanzhi Liu, Sen Luo, Yutian Lei, Jianhua Wu, Zhuo Huang, Kunzheng Wang, Pei Yang, Xin Huang

**Affiliations:** a Bone and Joint Surgery Center, Second Affiliated Hospital of Xi’an Jiaotong University, Xi’an, China; b Department of Cardiovascular Medicine, First Affiliated Hospital of Xi’an Jiaotong University, Xi’an, China

**Keywords:** Machine learning, metabolic syndrome, bioinformatics, biomarkers, gene hub

## Abstract

Early risk assessments and interventions for metabolic syndrome (MetS) are limited because of a lack of effective biomarkers. In the present study, several candidate genes were selected as a blood-based transcriptomic signature for MetS. We collected so far the largest MetS-associated peripheral blood high-throughput transcriptomics data and put forward a novel feature selection strategy by combining weighted gene co-expression network analysis, protein-protein interaction network analysis, LASSO regression and random forest approaches. Two gene modules and 51 hub genes as well as a 9-hub-gene signature associated with metabolic syndrome were identified. Then, based on this 9-hub-gene signature, we performed logistic analysis and subsequently established a web nomogram calculator for metabolic syndrome risk (https://xjtulgz.shinyapps.io/DynNomapp/). This 9-hub-gene signature showed excellent classification and calibration performance (AUC = 0.968 in training set, AUC = 0.883 in internal validation set, AUC = 0.861 in external validation set) as well as ideal potential clinical benefit.

## 1. Introduction

Metabolic syndrome (MetS) is a complex abnormality with several components, such as insulin resistance, diabetes, obesity, hypertension, and hyperlipidemia [1,2]. The occurrence and development of MetS and its components are always associated with poor cardiovascular outcomes, especially for individuals with obesity and insulin resistance, which are the core pathophysiological features of MetS [3–5]. The lack of effective risk assessment biomarkers makes early intervention for MetS and MetS-related diseases difficult [6,7]. Studies have reported potential biomarkers of MetS; however, there is still a lack of definitive clinical risk assessment biomarkers [8,9]. Research on MetS biomarkers is limited to genomics, and the association between MetS and single nucleotide polymorphisms (SNPs) [10,11]. Few studies have focused on MetS-specific biomarkers from a transcriptomics perspective [12].

In high-throughput transcriptomics, microarrays and next-generation sequencing (NGS) have been widely used to measure RNA expression levels [13–15]. In addition, advanced bioinformatics approaches, such as weighted gene co-expression network analysis (WGCNA), can play an important role in the identification of disease biomarkers, as they have high sensitivity, specificity, and efficiency, based on high-throughput transcriptomic data [16,17]. Compared to traditional bioinformatics methods, such as differentially expressed gene (DEG) analysis, network-focused algorithm WGCNA can establish a weighted scale-free co-expression network, and then identify key gene modules and hub genes [18]. Machine learning (ML), as a key aspect of artificial intelligence, has been increasingly applied in many biomedical fields, such as biomarker identification, diagnosis signature development, and drug target discovery [19,20]. Moreover, some ML methods, such as least absolute shrinkage and selection operator (LASSO) regression and random forest (RF), can significantly improve biomarker development for multifactorial and complicated diseases [21,22].

In this study, an integrated bioinformatic approach using WGCNA was performed on the largest MetS-associated peripheral blood high-throughput transcriptomic data set. Several hub genes were identified via protein–protein interaction (PPI) network analysis, and further hub gene feature selection was conducted by combining LASSO regression and RF algorithms. Finally, a logistic regression and a web nomogram calculator for MetS risk (https://xjtulgz.shinyapps.io/DynNomapp/) was established based on the training set, and the diagnostic value of selected hub gene features was measured using internal and external validation data. To further detect the differences in hub gene expression in peripheral blood and plasma, NGS was carried out in plasma samples of MetS patients and a control group (healthy patients). The current study aimed to identify gene parameters with high diagnostic value and clinical implications for MetS, using comprehensive bioinformatics and ML feature selection methods. This study provides a novel strategy for more effective and reliable biomarker development.

## 2. Materials and methods

### 2.1. Data collection and preprocessing

MetS causes highly specific gene expression changes in peripheral blood. Public gene expression datasets based on peripheral blood samples containing MetS-associated clinical diagnosis information were collected from the Gene Expression Omnibus database (GEO database; http://www.ncbi.nlm.nih.gov/geo/). The training set consisted of 70% of samples randomly selected from the GSE152073 (n = 90) and GSE98895 (n = 40) combined datasets (gene expression microarray data of peripheral blood), and the remaining 30% was used as internal validation data [23,24]. GSE124534 (n = 17, gene expression microarray data of peripheral blood) was used for external validation [25]. Subjects diagnosed with other metabolic diseases or acute trauma, such as osteoporosis or femoral neck fracture, which may cause gene expression changes, were excluded. Detailed information on these datasets is listed in **Supplementary Table 1**. After removing the outliers and probes that were duplicate or could not be annotated, gene expression data were normalized and batch effects removed using the ‘limma’ package in R. Missing data was imputed using the R software package ‘impute.’

### 2.2. WGCNA

WGCNA was performed based on GSE98895 datasets using the R package ‘WGCNA’ [26]. First, the Pearson’s correlation was calculated for all pairs of genes to establish a similarity matrix. Second, an appropriate soft-thresholding power of two was selected to meet the scale-free topology (scale-free R2 > 0.9) criterion using the function ‘pickSoftThreshold.’ Third, a topological overlap matrix and corresponding dissimilarity matrix were constructed. Then, the ‘blockwiseModules’ function was run with the following major parameters: maxBlockSize = 5000, minModuleSize = 30, and mergeCutHeight = 0.25. Several gene modules were identified through hierarchical clustering with a dynamic tree-cutting algorithm. Finally, the correlation between gene modules and clinical phenotypes was calculated to identify clinically significant modules.

### 2.3. Enrichment analysis of modules

To explore the function and signaling pathways associated with these modules, Gene Ontology (GO) function enrichment analysis and Kyoto Encyclopedia of Genes and Genomes (KEGG) pathway enrichment analysis were performed, as well as Gene Set Enrichment Analysis (GSEA) using the ‘clusterProfiler,’ ‘enrichplot,’ ‘DOSE,’ and ‘ggplot2’ packages in R software [27]. A P value of <0.05 was set as the threshold.

### 2.4. PPI network construction and hub gene identification

A protein–protein interaction (PPI) network was constructed based on the STRING database (Search Tool for the Retrieval of Interacting Genes, version 11.0, combined score >0.4). Connectivity degrees in the network were then calculated, and the top 5% of genes with the highest connectivity degree were identified as hub genes for further analysis. Visualization of hub genes in the PPI network was achieved using Cytoscape software (version 3.7.0).

### 2.5. Clinical plasma sample collection

Peripheral blood samples were obtained from five patients with MetS and five healthy volunteers from the First Affiliated Hospital of Xi’an Jiaotong University, defined using the World Health Organization’s MetS definition. MetS diagnosis can be made based on the presence of impaired fasting glucose, impaired glucose tolerance, type 2 diabetes mellitus (T2DM) or insulin resistance, and two or more of the following [1]: waist-to-hip ratio > 0.90 in men; waist-to-hip ratio > 0.85 in women, and/or body mass index > 30 kg/m^2^ [2]; serum triglyceride level ≥ 1.7 mmol/L [3]; HDL cholesterol < 0.9 mmol/L in men, < 1.0 mmol/L in women, or treatment for dyslipidaemia [4]; blood pressure ≥ 140/90 mm Hg; and [5] microalbuminuria [28]. This study was approved by the Ethics Committee of the First Affiliated Hospital of Xi’an Jiaotong University (Ethical Approval number: XJTU1AF2019LSL-014). All participants provided written informed consent in advance.

### 2.6. RNA extraction and high-throughput sequencing

Total RNA was extracted from plasma samples using TRIzol LS Reagent (Invitrogen), according to the manufacturer’s instructions. Sequencing libraries were generated using the NEBNext Poly(A) mRNA Magnetic Isolation Module (New England Biolabs), RiboZero Magnetic Gold Kit (Epicenter, Illumina Company), and KAPA Stranded RNA-Seq Library Prep Kit (Illumina). An Agilent Bioanalyzer 2100 system (Agilent) was used to qualify the sequencing libraries. Finally, high-throughput NGS was carried out using the TruSeq SR Cluster Kit (Illumina), based on the Illumina HiSeq 4000 sequencing platform (Illumina). The sequencing data has been uploaded to ArrayExpress database (E-MTAB-10494) .

### 2.7. Plasma mRNA differential expression analysis

Trimmed reads were identified after raw sequencing data quality control and filtering using the Solexa pipeline program (version 1.8) and Cutadapt software. Subsequently, human reference genome indexing (hg38) was obtained using Bowtie (http://bowtie-bio.sourceforge.net/index.shtml). Sequence alignment was performed using the Hisat2 program. The R package ‘edgeR’ was used to detect DEGs [29]. The threshold for DEGs was set as |log2FC|≥ 1 and P value < 0.05.

### 2.8. Hub gene feature selection strategy

ML algorithms are more powerful than traditional methods for complex classification, like medical diagnosis and treatment. In this study, two ML approaches: LASSO regression and RF using R packages ‘glmnet’ and ‘randomForest’ were combined to achieve feature selection [30]. The feature selection was cross-checked, and several hub genes were selected according to the classification accuracy. Hub genes from LASSO regression and RF feature selection were further used to establish a diagnosis classifier.

### 2.9. Web nomogram calculator construction and validation of a nine-hub-gene signature

The R package ‘rms’ was used to establish a logistic regression model, based on expression data in the training set. A corresponding web nomogram calculator for MetS risk was constructed to visualize the diagnostic effect of the selected hub gene signature. Internal and external validations were then performed to determine the web nomogram calculator performance. The area under curve (AUC) value of the receiver operating characteristic (ROC) curve was calculated using the ‘pROC’ package in R, which can depict the classification ability [31]. The Hosmer–Lemeshow goodness-of-fit test and a calibration curve analysis were conducted to indicate the calibration. In addition, a decision curve analysis was carried out using the ‘rmda’ package to evaluate the clinical application value and net benefit of the nomogram.

## 3. Results

In this study, through combined integrated bioinformatic approaches and machine learning algorithms, we identified a nine-hub-gene signature with high diagnostic value and clinical implications for MetS. Besides, current work provides a novel strategy for more effective and reliable biomarker development.

### 3.1. WGCNA construction and identification of key modules

**Figure 1.**
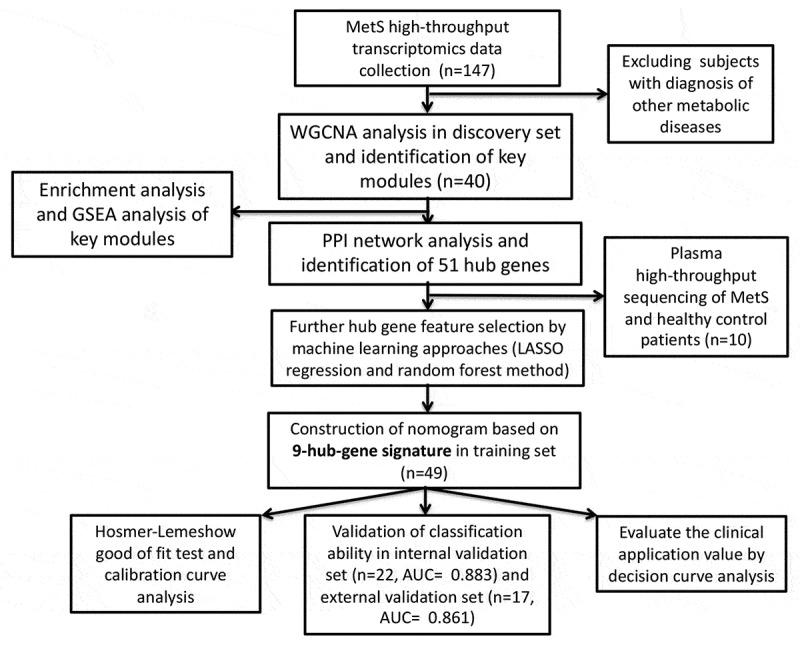
Flow chart of data processing and analysis

**Figure 2.**
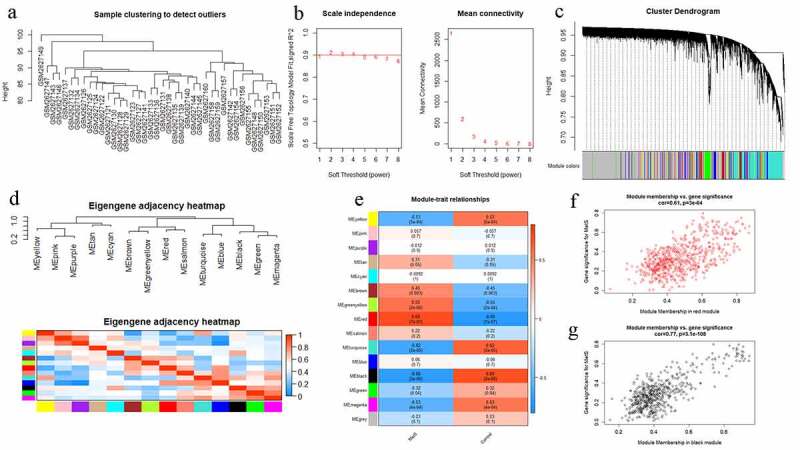
Weight gene correlation network analysis (WGCNA). (a) Sample clustering dendrogram and outliers detection. (b) Selection of the soft threshold. Scale-free topology fitting index R2 analysis (left) and mean connectivity for various soft threshold powers (right). The red line in the left panel means R2 = 0.9. (c) Clustering diagram of gene modules represented by different colors. (d) Clustering tree of gene modules and the correlation heatmap of the module eigengenes. (e) Heatmap of the relationship between modules and MetS: red for positive correlation and blue for negative correlation. (f,g) Scatter diagrams of genes in red module and black module. X-axis represents gene significance and y-axis represents module membership

The workflow of this study is shown in Figure 1. The most comprehensive sets of MetS-associated high-throughput transcriptomic data from the GEO database were combined (**Supplementary Table 1)**. Gene expression profiles from GSE98895 were used to perform WGCNA. After preprocessing and batch effect removal, 25,148 gene expression data were identified from peripheral blood samples from 20 MetS and 20 control patients. Sample-clustering analysis, based on Pearson’s correlation and average linkage approaches, showed no outliers (Figure 2a). To achieve scale-free topology (scale-free R2 > 0.9), a soft-thresholding power β = 2 was selected (Figure 2b). Subsequent WGCNA network construction and average linkage hierarchical clustering detected 14 gene modules. Detailed hierarchical clustering information is shown in Figure 2c,d. The correlation analysis between these modules and MetS showed that the red module (618 genes) and black module (546 genes) were highly associated with MetS (Figure 2e). Hence, these two modules were identified as the key modules of MetS for further analysis. Scatter diagrams containing key module GS and MM information are shown in Figure 2f,g.

### 3.2. GO and pathway enrichment analysis

**Figure 3.**
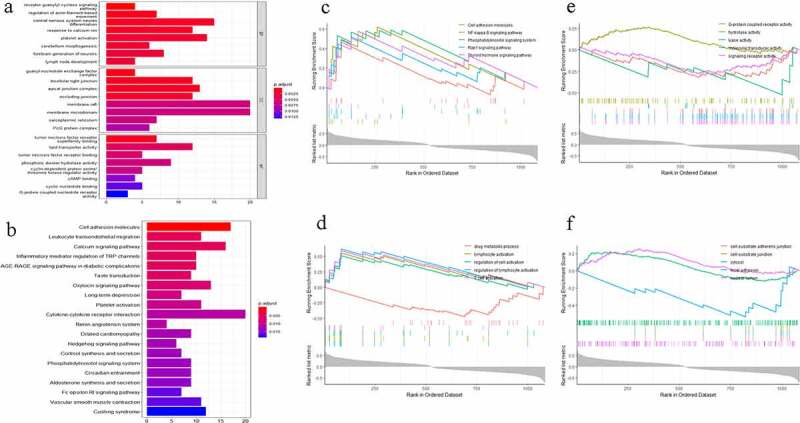
(a) Enrichment analysis of Gene Ontology (GO) function. (b) Enrichment analysis of Kyoto Encyclopedia of Genes and Genomes (KEGG) signaling pathway. The color represents the P value and X-axis represents gene number. (c) Gene Set Enrichment Analysis (GSEA) of KEGG signaling pathway. (d) Gene set enrichment analysis of biology process (BP). (e) Gene set enrichment analysis of molecular function (MF). (f) Gene set enrichment analysis of cellular component (CC)

The GO functional enrichment analysis showed that the MetS-associated genes in red and black modules were mainly enriched in biological processes (BP), such as the receptor guanylyl cyclase signaling pathway, central nervous system neuron differentiation, response to calcium ion, and platelet activation. In addition, these genes were associated with molecular functions (MF), such as tumor necrosis factor receptor and lipid transporter activity. Cellular components (CC), such as cellular junctions and guanyl-nucleotide exchange factor complexes, may correlate with the development of MetS. The KEGG signaling pathway enrichment analysis indicated that these genes were significantly enriched in signaling pathways, such as cell adhesion molecules, leukocyte transendothelial migration, and the calcium signaling pathway (Figure 3a,b). In addition, GSEA further revealed the function and signaling pathways of these genes, and showed a similar result to CC GO and KEGG pathway enrichment analysis. BP GSEA and MF GSEA suggested that BP, such as regulation of lymphocyte activation, drug metabolic processes, and MF, such as lyase activity, hydrolase activity, molecular transducer activity, and G-protein coupled receptor activity, might be involved in the development of MetS (Figure 3c-f).

### 3.3. PPI network construction and hub gene identification

**Figure 4.**
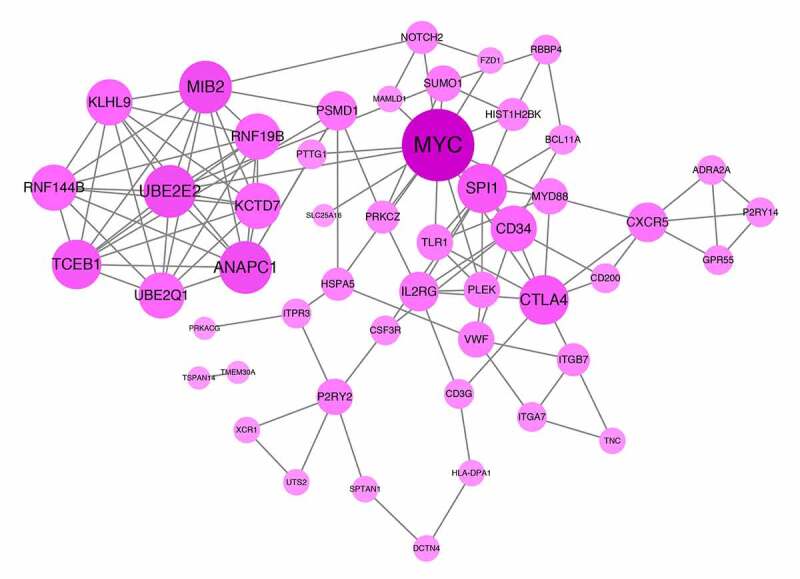
Protein-protein interaction (PPI) network. The gradual color and spot size represents the connectivity degree

PPI networks were established using the STRING database, based on genes in the red and black modules. The degree of connectivity was calculated, and the top 5% of genes (51 genes) with the highest connectivity were selected as hub genes associated with MetS. Hub genes with a high degree of connectivity, such as *MYC, UBE2E2, MIB2, ANAPC1, TCEB1, CTLA4*, and *SPI1*, might play important roles in the development of MetS, and could serve as potential biomarkers and therapeutic targets. The visualization of the hub gene PPI network is shown in Figure 4. These 51 hub genes (**Supplementary Table 2**) were used for further feature reduction analysis and model construction.

### 3.4. Hub gene expression level in plasma

12,954 genes were identified in plasma samples from five patients in the MetS group and five patients in the control group, and 45 upregulated and 186 downregulated DEGs were identified in the MetS group compared to the control **(Supplementary Table 3)**. The plasma expression of the 51 hub genes did not differ significantly between MetS patients and healthy controls (**Supplementary Table 4)**. These results indicate that the potential function and diagnostic value of these 51 hub genes in peripheral blood should be determined, instead of in plasma components. This outcome defines the sampling type for further noninvasive MetS screening or diagnostic tools.

### 3.5. Novel hub gene feature selection strategy

**Figure 5.**
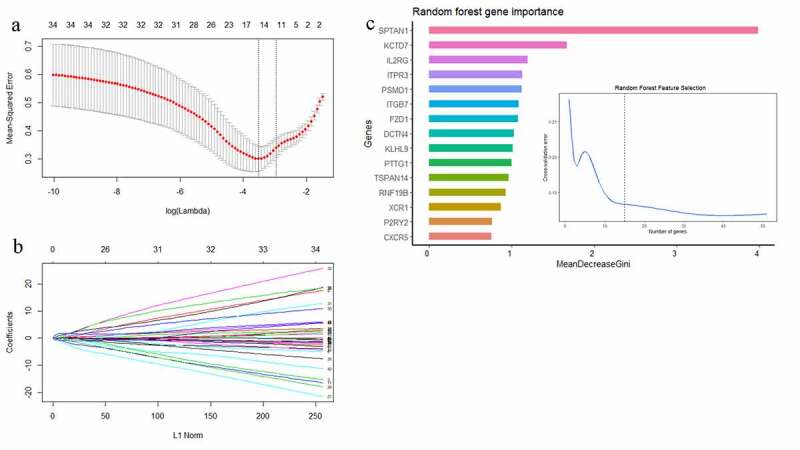
(a) The mean-squared error of LASSO regression. Y-axis represents mean-squared error. X-axis represents the ideal gene feature amount on various of lambda value. Left dotted line means the minimum of mean-squared error and the right dotted line means one standard deviation above minimum of mean-squared error. (b) Coefficients distribution trend of LASSO regression. (c) The importance of hub gene features based on random forest algorithm and the ideal gene feature amount

In this study, LASSO regression analysis and RF were used for feature selection. The expression data of the 51 hub genes were entered into LASSO regression models, and a 10-fold cross-validation was performed to detect the optimal classification accuracy (Figure 5a,b). Hence, 15 hub gene features were obtained based on LASSO regression analysis, including *ADRA2A, CXCR5, FZD1, HLA.DPA1, HSPA5, KCTD7, KLHL9, P2RY14, P2RY2, PRKACG, PSMD1, PTTG1, REEP4, SPTAN1, and TSPAN14*. In addition, an RF model was constructed using the expression profiles of the 51 hub genes, and the classification importance of hub gene features was measured by the decrease in the Gini coefficient (MeanDecreaseGini). Fifteen hub gene features were chosen using an RF approach, comprising *SPTAN1, KCTD7, IL2RG, ITPR3, PSMD1, ITGB7, FZD1, DCTN4, KLHL9, PTTG1, TSPAN14, RNF19B, XCR1, P2RY2, and CXCR5* (Figure 5c). Finally, the results of these two gene feature selection methods were combined by taking the intersection, and nine-hub-gene features (*SPTAN1, KCTD7, PSMD1, FZD1, KLHL9, PTTG1, TSPAN14, P2RY2, and CXCR5*) were selected for further analysis. Based on Human Protein Atlas database, the mRNA blood cell type distribution and protein concentration in plasma of these nine hug genes were showed in **Supplementary Table 5**.

### 3.6. Web nomogram calculator construction and validation of nine-hub-gene signature

**Figure 6.**
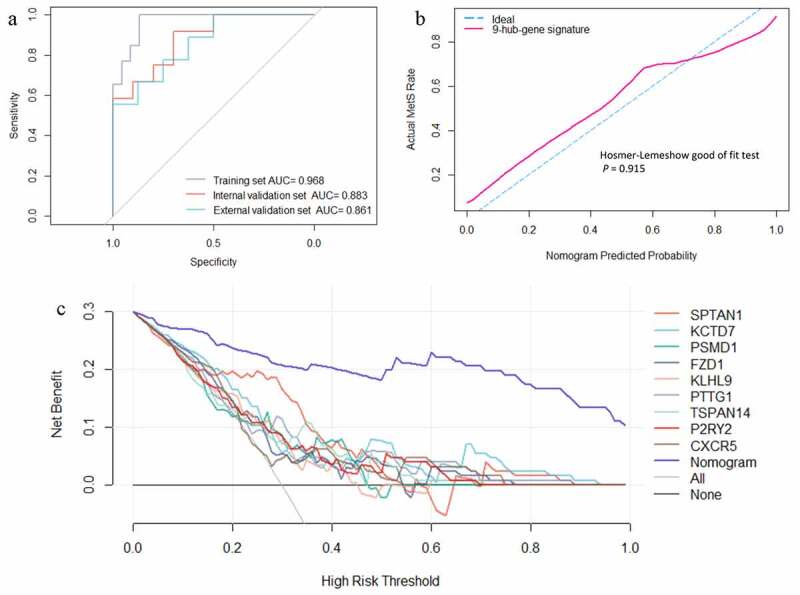
(a) Receiver operating characteristic curves of the web nomogram calculator based on the 9-hub-gene signature. (b) Calibration curve analysis and Hosmer-Lemeshow good of fit test of the web nomogram calculator based on the 9-hub-gene signature. (c) Decision curve analysis of every single gene feature and the web nomogram calculator based on the 9-hub-gene signature

The expression profiles of the nine selected gene features were entered into a logistic regression, and then, to validate the diagnostic value of this nine-hub-gene signature, a web nomogram calculator for MetS risk was established based on the training set (https://xjtulgz.shinyapps.io/DynNomapp/). The ROC curve analysis (Figure 6a) showed that this MetS diagnostic nomogram had excellent classification ability (AUC = 0.968 in training set, AUC = 0.883 in internal validation set, AUC = 0.861 in external validation set). The ROC curves of every hub gene are shown in Supplementary Figure 1. In addition, a calibration curve analysis was performed, and the Hosmer–Lemeshow goodness-of-fit test (*P*= 0.915) showed good calibration of this nomogram (Figure 6b). Furthermore, the decision curve plotted the standardized net benefit of the MetS diagnostic nomogram for different decision thresholds (Figure 6c). These results indicate that the application of this MetS diagnostic nomogram can lead to ideal diagnostic outcomes.

## 4. Discussion

Recently, considerable amount of research has been conducted on MetS; however, early diagnosis and intervention remains difficult because of a lack of effective biomarkers and targeted treatment [32]. To the best of our knowledge, this is the first study to identify a key gene module and 51 MetS-associated hub genes by combining a WGCNA bioinformatics approach and PPI network analysis. Genes in this key module were mainly enriched in signaling pathways, such as cell adhesion, leukocyte transendothelial migration signaling, nuclear factor kappa B (NF-κB), and functions such as lymphocyte activation. These 51 hub genes may play important roles in the development of MetS. Cheung et al. suggested that *MYC* (MYC proto-oncogene) can serve as an important mediator of impaired insulin secretion and β-cell apoptosis [33]. Some studies have indicated that the SNPs in *UBE2E2* (ubiquitin conjugating enzyme E2) are associated with the development of T2DM [34,35]. Additionally, mindbomb E3 ubiquitin protein ligase 2 (*MIB2*), anaphase promoting complex subunit 1 (*ANAPC1*), and *ELOC*, Elongin C (*TCEB1*), are also involved in ubiquitination, which can affect the development of insulin resistance and MetS [36,37]. Cytotoxic T-lymphocyte associated protein 4 (*CTLA4*) is involved in T-cell immune responses, and thus, it regulates the pathogenesis of insulin resistance and insulin-dependent diabetes mellitus [38,39]. Moreover, the upregulation of Spi-1 proto-oncogene (*SPI1*, or *PU.1*) in adipocytes can cause insulin resistance by stimulating reactive oxygen species production and inflammatory cytokine gene expression [40,41]. These hub genes could serve as biomarkers for MetS and many of their contributing components.

Through ML feature selection methods, a nine-hub-gene signature with high diagnostic value and clinical implications for MetS was obtained. Dhana et al. found that the proteasome 26S subunit, non-ATPase (*PSMB1*) gene was associated with both body mass index and waist circumference, and could serve as a biomarker for obesity-related diseases [42]. Some studies have shown that frizzled class receptor 1 (*FZD1*) is related to insulin resistance [43,44]. In addition, Kelch-like family member 9 (*KLHL9*) can induce insulin resistance by regulating insulin receptor substrate-1 (*IRS1*) degradation [45]. Pituitary tumor-transforming gene 1 (*PTTG1*) is a crucial factor in the development and physiological responses of pancreatic beta-cells, and its dysregulation can result in diabetes [46]. Tetraspanin 14 (*TSPAN14*) can interact with ADAM metallopeptidase domain 10 (*ADAM10*) and then regulate leukocyte development and inflammatory immunity function [47]. Previous studies have demonstrated that purinergic receptor (*P2Y2*) contributes to the development of chronic high-fat diet-induced metabolic dysfunction and insulin resistance [48,49]. Furthermore, *P2RY2* is involved in the process of immune cell infiltration in MetS [50]. Follicular helper T-cells (Tfh) of diabetic patients express elevated levels of C-X-C motif chemokine receptor 5 (*CXCR5*), and there is a dysregulation of circulating CD4+ CXCR5 + T-cells in diabetes patients [51,52]. These results indicated nine-hub-gene signature is highly associated with MetS.

Finally, the classification ability, calibration, and potential clinical benefit of the blood-based, nine-hub-gene signature was verified in internal and external validation sets. Previous studies have not investigated the diagnostic value of these nine hub genes for MetS; an early screening or diagnostic tool for MetS has not been developed [53]. However, in this study, the blood-based, nine-hub-gene signature combined with logistic regression and visualized as a nomogram produced an excellent classification and calibration performance. The AUC of the ROC curves reached 0.883 in the internal validation set and 0.861 in the external validation set. The Hosmer–Lemeshow goodness-of-fit test (*P* = 0.915) showed good calibration. A further decision curve analysis showed that this nomogram has a better net benefit than any single gene signature in almost all decision threshold ranges. Overall, these results indicate that this nine-hub-gene signature is useful for MetS-associated, blood-based risk assessments in clinical applications.

In this study, the largest MetS-associated peripheral blood high-throughput transcriptomics dataset was collected. However, further large, independent patient cohort validation studies are still needed to establish a diagnostic model for clinical applications.

## 5. Conclusion

Because of its excellent classification ability, calibration, and potential clinical benefits, the nine-hub-gene signature identified in the present study can be used to accurately assess MetS risk. In addition, a novel risk assessment biomarker selection method is proposed by combining WGCNA approaches, PPI network analysis, LASSO regression, and RF feature selection algorithms. In addition, high-throughput sequencing was performed to detect the plasma cell-free mRNA expression level in MetS patients compared with healthy controls, which can provide a reliable basis for sampling type in MetS risk assessment.

## Supplementary Material

Supplemental Material

## Data Availability

The data that support the findings of the this study are available from the corresponding author on reasonable request. The datasets for this study can be found in the Gene Expression Omnibus (GEO) database [https://www.ncbi.nlm.nih.gov/geo/].

## Research highlights

1. Combining bioinformatics analysis and machine learning algorithms
2. Providing a novel strategy for biomarker identification
3. A nine-hub-gene signature with high diagnostic value for MetS
