# Supplementary figures and images for: A nine-hub-gene signature of metabolic syndrome identified using machine learning algorithms and integrated bioinformatics
Source: Bioengineered. 2021 Sep 13;12(1):5727–38. doi: 10.1080/21655979.2021.1968249 (PMC8806918; doi:10.1080/21655979.2021.1968249)

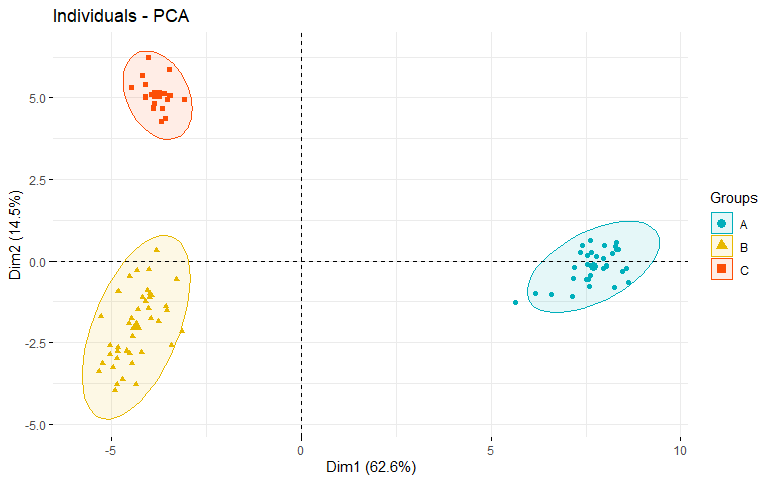

Supplement: Supplemental Material [file KBIE_A_1968249_SM2213.zip › suppl/Supplementary Figure 2.tiff]

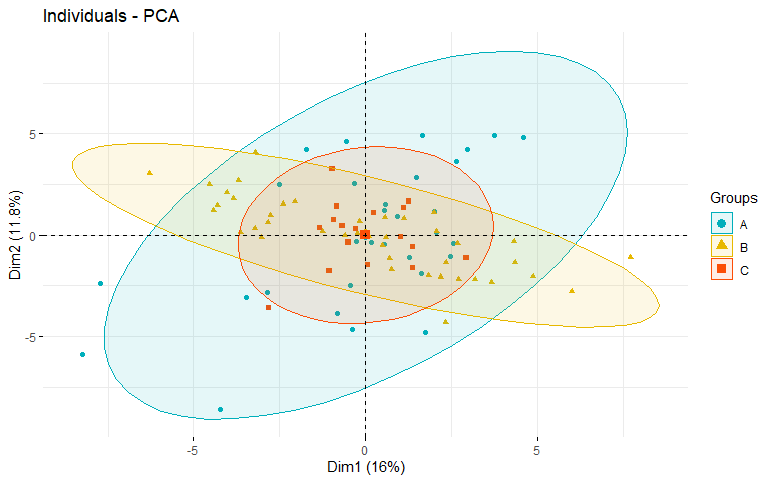

Supplement: Supplemental Material [file KBIE_A_1968249_SM2213.zip › suppl/Supplementary Figure 3.tiff]
